# Differences between* Mycobacterium*-Host Cell Relationships in Latent Tuberculous Infection of Mice* Ex Vivo* and Mycobacterial Infection of Mouse Cells* In Vitro*

**DOI:** 10.1155/2016/4325646

**Published:** 2016-03-15

**Authors:** Elena Ufimtseva

**Affiliations:** The Research Institute of Biochemistry, 2 Timakova Street, Novosibirsk 630117, Russia

## Abstract

The search for factors that account for the reproduction and survival of mycobacteria, including vaccine strains, in host cells is the priority for studies on tuberculosis. A comparison of BCG-mycobacterial loads in granuloma cells obtained from bone marrow and spleens of mice with latent tuberculous infection and cells from mouse bone marrow and peritoneal macrophage cultures infected with the BCG vaccine* in vitro* has demonstrated that granuloma macrophages each normally contained a single BCG-*Mycobacterium*, while those acutely infected* in vitro* had increased mycobacterial loads and death rates. Mouse granuloma cells were observed to produce the IFN*γ*, IL-1*α*, GM-CSF, CD1d, CD25, CD31, СD35, and S100 proteins. None of these activation markers were found in mouse cell cultures infected* in vitro* or in intact macrophages. Lack of colocalization of lipoarabinomannan-labeled BCG-mycobacteria with the lysosomotropic LysoTracker dye in activated granuloma macrophages suggests that these macrophages were unable to destroy BCG-mycobacteria. However, activated mouse granuloma macrophages could control mycobacterial reproduction in cells both* in vivo* and in* ex vivo* culture. By contrast, a considerable increase in the number of BCG-mycobacteria was observed in mouse bone marrow and peritoneal macrophages after BCG infection* in vitro*, when no expression of the activation-related molecules was detected in these cells.

## 1. Introduction

At present, tuberculosis (TB) remains to be one of the world's most serious health concerns. In 2014, the WHO reported 9 million new TB cases and about 1.5 million deaths [1]. The increasing incidence of infection with antibiotic-resistant strains of* Mycobacterium tuberculosis* is an alarming trend of recent years [1–3]. This is indicated by an increasing incidence of acutely progressing forms of drug-resistant TB with severe clinical manifestations and a widespread occurrence of the pathological process in the organism [2–4]. In 2014, 480,000 new cases of ТВ with multiple drug resistance were diagnosed, of which only 48% recovered [1]. At present, there is the only anti-TB vaccine called the “Bacillus Calmette-Guérin” (BCG) prepared from an attenuated live strain of* M. bovis*, a pathogen in cattle. The BCG vaccine is considered to be only effective against disseminated forms of TB in children, but why it does not protect adults against pulmonary TB is not yet known [5, 6].

Man normally becomes infected with* M. tuberculosis* by aerosol transmission. Pulmonary macrophages entrap mycobacteria by phagocytosis and destroy them in phagolysosomes using active forms of oxygen and nitrogen, lysosomal hydrolases, and toxic peptides in a low-pH medium. The proinflammatory cytokines IFN*γ*, IL-1*α*, and GM-CSF secreted by activated cells of different types exert regulation on immune processes and inflammation in response to invasion by pathogens, controlling the development of infection in the organism by enhancing the microbicidal activity of the cells of the immune system [4, 6]. The uncontrollable replication of mycobacteria in the human organism normally results in acute TB; however, activation of the innate and adaptive immune responses in the human organism most often leads to latent, asymptomatic ТВ infection. According to the WHO, one-third of the world's population is a latent carrier of* M. tuberculosis* in chronic granulomatous inflammatory lesions largely composed of macrophages [2, 5, 6]. Low BCG-mycobacterial loads in animal organs and tissues at different time points of chronic infection had previously been established by bacteriological methods in a model of latent tuberculous infection under which mice were infected with BCG-mycobacteria* in vivo* [7–10]. Using our original model of mouse granulomas in* ex vivo* culture, we have, for the first time, determined the bacterial load in macrophages, dendritic cells, and multinucleate Langhans giant cells in separate granulomas obtained from mice with latent tuberculous infection after* in vivo* exposure to BCG vaccine [11, 12]. In some host cells, not only did BCG-mycobacteria survive, but also they were actively reproducing and formed cording colonies, cording being the indication of their virulence [12]. Interestingly, there was a difference in behavior between mycobacteria of virulent and nonvirulent strains in* in vitro* cultures of infected human, mouse, and cow cells [13–18]. Mycobacteria of virulent strains were actively reproducing in cells infected* in vitro*, while attenuated mycobacteria of the BCG vaccine and* M. tuberculosis* of nonvirulent strains were basically found in vacuoles before they were destroyed there within 2–7 days of observation* in vitro* [15]. However, there are very few comparative studies of relationships between mycobacteria of different strains and host cells in animals infected* in vivo* or following acute infection* in vitro* [19, 20]. And very few are the studies researching relationships between BCG-mycobacteria and host cells [11, 12, 19, 21]. As is known, BCG vaccines can occasionally cause severe disease in children with inborn errors of immunity often referred to as BCG-osis [22, 23]. Importantly, clinical observations of BCG infection (including BCG adenitis) in AIDS patients after as many as 30 years following BCG vaccination are still being discussed [6]. Therefore, understanding relationships between BCG-mycobacteria and host cells both after infection* in vivo* and after acute infection* in vitro* is important for studying the development of BCG-induced anti-TB immunity, developing better BCG-based vaccines [5, 6], and testing vaccine candidates in animal models [24], including mouse models of tuberculous and nontuberculous mycobacterial infections [24, 25].

In the present work, we conducted a comparative study of the mycobacterial loads in granuloma cells from the bone marrow and spleens of mice with latent tuberculous infection following infection with BCG* in vivo* and several days of* ex vivo* culture and in the cultures of bone marrow cells and peritoneal macrophages obtained from intact mice and infected with BCG* in vitro*. As a result, we observed a considerable increase in the number of mycobacteria in the macrophages of bone marrow cell cultures and peritoneal macrophages within 120 h following BCG infection* in vitro* and the death of cells having increased BCG loads. Throughout 48–120 h of* ex vivo* culture, mouse granuloma macrophages each basically remained to contain a single BCG organism, and increased numbers of such microorganisms in some macrophages did not cause the host cells to die. Analysis of the levels of the proinflammatory cytokines IFN*γ* and IL-1*α*, the growth factor GM-CSF, and various cell-surface markers in BCG-containing macrophages in* ex vivo* and* in vitro* cultures suggested that although the active production of these molecules in mouse granuloma cells did not help in eliminating all mycobacteria in the host cells, it helped in restricting mycobacterial reproduction in granuloma macrophages. By contrast, a considerable increase in the number of BCG-mycobacteria was observed in those* in vitro* infected mouse bone marrow and peritoneal macrophages, whether alive or dead by apoptosis/necrosis, in which no active synthesis of these markers was going on.

## 2. Materials and Methods

### 2.1. Animals

Two-month-old BALB/c male mice were obtained from the Animal Breeding Facility of the Institute of Cytology and Genetics of the Siberian Branch of the Russian Academy of Sciences (Novosibirsk, Russia). Mice were bred and maintained under standard vivarium conditions, with water and food provided* ad libitum*. Animal experiments were conducted in accordance with “The Guidelines for Manipulations with Experimental Animals” issued by the Russian Ministry of Health (guideline 755). All experimental procedures were approved by the Local Ethical Committee of the Research Institute of Biochemistry (Novosibirsk, Russia).

### 2.2. Infection of Mice

Mice were infected with a vaccine prepared from an attenuated live strain of* M. bovis* (the Bacillus Calmette-Guérin vaccine, BCG-1, the Institute of Microbiology and Epidemiology, Moscow, Russia) at a dose of 0.5 mg per mouse, which amounted to 3 × 10^6^ viable BCG-mycobacteria in 0.9% NaCl solution. Twenty-four mice were each infected via tail vein injection with 100 *μ*L of the suspension and four mice were each infected intraperitoneally with 200 *μ*L of the suspension.

### 2.3. Isolation and* Ex Vivo* Culture of Mouse Granulomas

Mice were anesthetized and killed by cervical dislocation. Isolation of granulomas from the spleens and bone marrow of mice after 20 days, one month, and two months following infection was performed as previously described [11, 12]. Bone marrow was flushed from femurs with RPMI 1640 medium (BioloT, St. Petersburg, Russia). The spleens collected from the animals were cut into small pieces in 5 mL of RPMI 1640 medium with 50 *μ*g/mL gentamicin. Granulomas were isolated from the organ homogenates by centrifugation at 150 ×g at room temperature. The supernatants were removed. Granulomas were washed three times in 10 mL of RPMI 1640 medium with 50 *μ*g/mL gentamicin by centrifugation. The granuloma pellets were resuspended in the complete RPMI 1640 growth medium containing 10% heat-inactivated fetal bovine serum (FBS), 2 mM glutamine, and 50 *μ*g/mL gentamicin (BioloT, St. Petersburg, Russia), placed at low-medium density to 24-well tissue culture plates (Orange Scientific, Belgium) with glass coverslips at the bottom and cultured in 0.5 mL medium for several days at +37°C in an atmosphere containing 5% CO_2_. Granulomas were isolated from mice 1 and 2 on day 20 following intraperitoneal infection; from mice 1 and 2 ÷ 5 after one month following intraperitoneal and intravenous infection, respectively; from mice 1 ÷ 16 and 21 ÷ 24 after two months following intravenous infection; and from mouse 25 after two months following intraperitoneal infection. The mouse nomenclature used is explained [11, 12]. Peritoneal macrophages were isolated from mice 1 and 2 after 20 days and mouse 25 after two months following intraperitoneal infection and cultured under the same conditions as the granuloma cells. After isolation of granulomas from the bones of mice 1 and 2 after 20 days following infection [12], the other bone marrow cells from supernatants were cultured under the same conditions as the granuloma cells.

### 2.4. Cell Cultures and Infection* In Vitro*

Mice were anesthetized and killed by cervical dislocation. Peritoneal and bone marrow cells were isolated from four intact mice and used as separate mixtures of each cell culture. Peritoneal cells were isolated from peritoneal cavities in 7 mL of the complete RPMI 1640 growth medium. The femurs and tibias were removed and trimmed at both ends. Bone marrow was flushed out with 5 mL of RPMI 1640 medium containing 10% heat-inactivated FBS, 2 mM glutamine, and 50 *μ*g/mL gentamicin using a 22-gauge needle. Peritoneal macrophages and bone marrow cells (3 × 10^5^ mononuclear cells/well) were placed in 24-well tissue culture plates with glass coverslips at the bottom and cultured without stimulation in 0.5 mL of the complete growth medium for 24 h under the same conditions as the granuloma cells. After removal of growth medium with nonadherent cells, the monolayer cultures of bone marrow and peritoneal cells were washed twice with RPMI 1640 medium to prepare them for infection. Dry BCG vaccine was diluted in RPMI 1640 medium, passed several times through a 26-gauge needle to obtain stand-alone bacterial cells, and was added to the cells in some of the plate wells in the amount of 0.04 mg/well (2.4 × 10^5^ mycobacteria/well) in 200 *μ*L of the complete growth medium without antibiotics and incubated for 1 h at +37°C in an atmosphere containing 5% CO_2_. The cells were further washed twice with RPMI 1640 medium to remove extracellular bacteria and were cultured for various time periods under the same conditions as the granuloma cells. At hour 4 following BCG infection, more than 97% of peritoneal cells were found to be macrophages. At that time point, bone marrow cells were found to be precursor cells of different size, neutrophils, lymphocytes, and a few megakaryocytes. At hour 24 following BCG infection, the precursor cells differentiated into bone marrow macrophages both in the control (uninfected) cultures and in cell monolayers after 24 h of acute BCG infection* in vitro.* On day 3 after isolation of bone marrow cells, macrophages and some fibroblasts with only a few neutrophils were observed in these cultures. All macrophages in the cell cultures were CD14-positive after immunofluorescent staining for the CD marker (BD Biosciences, USA, 557896, data not shown). The control fibroblasts were obtained through spontaneous cell migration from fragments of the splenic capsule from intact mice in the wells of 24-well plates with cover glasses at the bottom with a minimal amount of complete growth medium at +37°C in an atmosphere containing 5% CO_2_. After removing tissue fragments, the migrant fibroblasts on the cover glasses were further cultured for an optimal density in 0.5 mL medium for several weeks. Three independent experiments on obtaining and studying fibroblasts, cells in the mouse bone marrow and peritoneal cultures have been conducted.

### 2.5. Cell Staining

At hours 48–120 of* ex vivo* culture, granuloma cells on coverslips were fixed with 4% formaldehyde solution in phosphate-buffered saline (PBS, pH 7.4) for 10 minutes at room temperature. The same procedure was applied to cells in bone marrow cell cultures and peritoneal macrophage cultures at hours 4, 24, 48, 72, 96, and 120 following infection with BCG* in vitro* and in control cell cultures. To visualize acid-fast mycobacteria within host cells and analyze their intracellular reproduction, the preparations were washed with PBS and stained by the Ziehl-Neelsen method, mycobacteria were counted under a microscope, and their mean number per macrophage was determined. The cells stained by the Ziehl-Neelsen method were further counterstained with 1% methylene blue.

The antibodies and reagents used for immunocytochemical and immunofluorescent staining were as follows: rabbit polyclonal primary antibodies to mycobacteria (Abcam, England, ab20832) diluted 1 : 200; rat monoclonal primary antibodies to mouse CD1d, CD25, CD31, CD35, CD86, GM-CSF, IFN*γ*, and CD11b (BD Biosciences, USA, 553843, 550529, 550274, 553816, 560582, 554404, 559065, and eBioscience, USA, 17-0112, resp.) diluted 1 : 50, 1 : 25, 1 : 50, 1 : 50, 1 : 100, 1 : 25, 1 : 100, and 1 : 250, respectively; hamster monoclonal primary antibodies to mouse CD80 and IL-1*α* (BD Biosciences, USA, 560526 and 550604, resp.) diluted 1 : 100 and 1 : 50, respectively; rabbit polyclonal primary antibodies to mouse fibronectin, vimentin, type I collagen (Abcam, England, ab23750, ab45939, Millipore, USA, AB765, resp.) diluted 1 : 500, 1 : 100, and 1 : 500, respectively, and to S100 proteins (courtesy of S. M. Sviridov of the Institute of Cytology and Genetics, Novosibirsk, Russia) diluted 1 : 50; biotin-conjugated goat polyclonal secondary antibodies to rat IgG (BD Biosciences, USA, 559286) diluted 1 : 50 and to rabbit IgG (Sigma, USA, B7389) diluted 1 : 100; mouse monoclonal biotin-conjugated secondary antibodies to hamster IgG (BD Biosciences, USA, 550335) diluted 1 : 50; FITC-labeled goat anti-rat IgG polyclonal secondary antibody (Abcam, England, ab6266) diluted 1 : 400; goat Alexa 488-conjugated anti-rabbit IgG secondary antibody (Invitrogen, USA, A11034) diluted 1 : 400; horseradish peroxidase-conjugated streptavidin (Sigma, USA, S5512); diaminobenzidine (Sigma, USA, D3939); Cy3-conjugated streptavidin (Sigma, USA, S6402) diluted 1 : 100; TRITC-labeled phalloidin to stain filamentous actin (Sigma, USA, P1951) diluted 1 : 100; and the acidotropic LysoTracker dye Red DND-99 (Invitrogen, USA, L7528).

In the experiments using LysoTracker Red DND-99, the cell preparations were incubated with 50 nM of the acidotropic dye for 5 minutes at +37°C in 5% CO_2_ before fixation. The cell preparations were fixed as described above, washed with PBS, permeabilized for 2 minutes with 0.3% Triton-X100 solution, blocked in PBS containing 2% BSA, and finally incubated first with rabbit polyclonal primary antibodies to mycobacteria and then with Alexa 488-conjugated goat polyclonal secondary antibodies to rabbit IgG.

Some of the fixed cell preparations were washed with PBS, blocked in PBS solution containing 2% BSA, and incubated first with rat monoclonal primary antibodies to mouse CD1d, CD25, CD31, and CD35, then with biotin-conjugated goat polyclonal secondary antibody to rat IgG, and finally with horseradish peroxidase-conjugated streptavidin in diaminobenzidine solution containing 0.05% H_2_O_2_. After incubation, the cells were counterstained with 1% methyl green. The antibodies against CD35 were labeled with biotin. Fluorescent visualization of CD35 was achieved using Cy3-conjugated streptavidin.

Some of the fixed cell preparations were washed with PBS, treated for 2 minutes with 0.3% Triton-X100 solution, blocked in PBS solution containing 2% BSA, and incubated with rat monoclonal primary antibodies to mouse IFN*γ* or rabbit polyclonal primary antibodies to mouse fibronectin, type I collagen, and S100 proteins. The cell preparations were further incubated with goat polyclonal biotin-conjugated secondary antibodies to rat IgG or rabbit IgG. Specific staining of the cell preparations was visualized using horseradish peroxidase-conjugated streptavidin in diaminobenzidine solution containing 0.05% H_2_O_2_. The cells were further counterstained with 1% methyl green.

Some of the fixed cell preparations were washed with PBS, blocked in PBS solution containing 2% BSA, and incubated with APC-conjugated rat monoclonal primary antibodies to mouse CD11b. After staining for CD, the cell preparations were washed with PBS, treated for 2 minutes with 0.3% Triton-X100 solution, and incubated with rabbit polyclonal primary antibodies to mouse vimentin, fibronectin, type I collagen, or S100 proteins. Fluorescent visualization of the proteins was achieved using Alexa 488-conjugated goat polyclonal secondary antibodies to rabbit IgG.

Some of the fixed cell preparations were washed with PBS, blocked in PBS solution containing 2% BSA, and incubated with rat monoclonal primary antibodies to mouse CD31, GM-CSF, or IFN*γ*. Fluorescent visualization of the proteins was achieved using FITC-labeled goat anti-rat IgG polyclonal secondary antibody. After visualization, the cell preparations were washed with PBS and incubated with rat monoclonal primary antibodies to mouse CD86, CD35, and CD11b, respectively. The antibodies against CD86, CD35, and CD11b were labeled with PE-Cy7, biotin, and APC, respectively. Fluorescent visualization of CD35 was achieved using Cy3-conjugated streptavidin.

Some of the fixed cell preparations were washed with PBS, treated for 2 minutes with 0.3% Triton-X100 solution, blocked in PBS solution containing 2% BSA, and incubated with TRITC-labeled phalloidin, to stain filamentous actin, and with rabbit polyclonal primary antibodies to mouse fibronectin or type I collagen. Fluorescent visualization of fibronectin and type I collagen was achieved using Alexa 488-conjugated goat polyclonal secondary antibody to rabbit IgG.

Some of the fixed cell preparations were washed with PBS, blocked in PBS solution containing 2% BSA, and incubated with hamster monoclonal primary antibody to mouse IL-1*α* and rat monoclonal primary antibodies to mouse CD1d or IFN*γ*. Fluorescent visualization of the proteins was achieved using FITC-labeled goat anti-rat IgG polyclonal secondary antibodies and biotin-conjugated mouse monoclonal secondary antibodies to hamster IgG and Cy3-conjugated streptavidin.

Some of the fixed cell preparations were washed with PBS, blocked in PBS solution containing 2% BSA, and incubated with PerCP-Cy5.5-conjugated hamster monoclonal primary antibodies to mouse CD80. After staining for CD, the cell preparations were washed with PBS, treated for 2 minutes with 0.3% Triton-X100 solution, and incubated with rat monoclonal primary antibodies to mouse IFN*γ*. Fluorescent visualization of cytokine was achieved using goat polyclonal FITC-labeled secondary antibody to rat IgG.

The cell preparations were incubated with the appropriate antibodies and streptavidin for 60 minutes at room temperature. The preparations of peritoneal macrophages obtained from normal uninfected BALB/c mice and cultured and fixed in the same manner as granuloma cells were used as the control groups. Fluorescent staining was analyzed using the VECTASHIELD Mounting Medium with DAPI (4′,6-diamidino-2-phenylindole) (Vector Laboratories, USA, H-1200). Confocal images of the cells were recorded; the preparations were washed in PBS for 20 minutes to remove the VECTASHIELD Mounting Medium and restained for acid-fast mycobacteria by the Ziehl-Neelsen method.

### 2.6. Microscopy

The cytological preparations were examined at the Shared Center for Microscopic Analysis of Biological Objects of the Institute of Cytology and Genetics, SB RAS, using an Axioskop 2* plus* microscope (Zeiss) and objectives with various magnifications (Zeiss), and photographed using an AxioCam HRc camera (Zeiss); the images were analyzed using the AxioVision 4.7 microscopy software (Zeiss). Cell preparations were stained with fluorescent dyes and examined under an LSM 780 laser scanning confocal microscope (Zeiss) using the LSM Image Browser and ZEN 2010 software (Zeiss). Granuloma cells were counted separately on each coverslip for each mouse in each test. In each experiment with bone marrow and peritoneal cell cultures, more than 1000 cells in 30 fields of view were analyzed at each time point.

### 2.7. Statistical Analysis

Statistical data processing was performed using MS Excel 2007 (Microsoft). Differences were tested for significance using Student's* t*-test.

## 3. Results

### 3.1. Mycobacterial Loads in Mouse Granuloma Cells at Different Time Points of* Ex Vivo* Culture and in Bone Marrow and Peritoneal Macrophages from Mice Infected with BCG* In Vitro*

**Figure 1:**
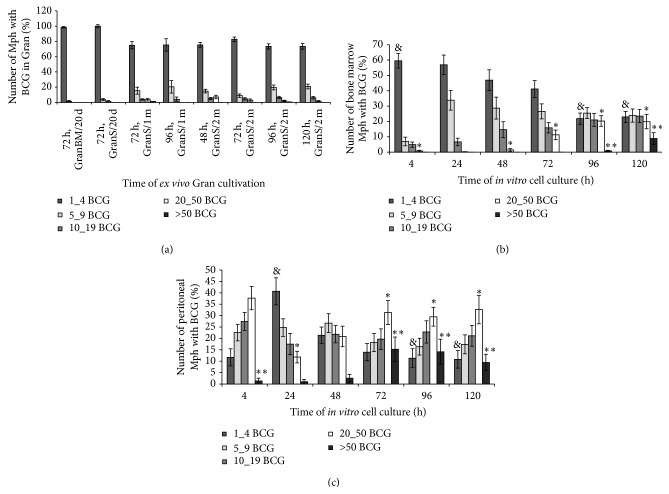
The number of macrophages (Mph) with different numbers of acid-fast BCG-mycobacteria (BCG) in them. (a) Macrophages in *n* granulomas (Gran) obtained from the bone marrow (BM/) and spleens (S/) of mice after 20 days (/20 d), one month (/1 m), and two months (/2 m) following infection with the BCG vaccine* in vivo* and after* ex vivo* culture for several hours. The number of macrophages with different numbers of BCG-mycobacteria in granulomas expressed as a percentage of the total number of infected macrophages in granulomas stained by the Ziehl-Neelsen (ZN) method. Data are expressed as the means ± SEM. At hour 48: GranS/2 m (3 mice, *n* = 111); at hour 72: GranBM/20 d (2 mice, *n* = 29), GranS/20 d (2 mice, *n* = 61), GranS/1 m (3 mice, *n* = 84), and GranS/2 m (5 mice, *n* = 133); at hour 96: GranS/1 m (1 mouse, *n* = 20) and GranS/2 m (4 mice, *n* = 103); at hour 120: GranS/2 m (4 mice, *n* = 52). (b) Bone marrow macrophages and (c) peritoneal macrophages with different numbers of BCG-mycobacteria following infection with the BCG vaccine* in vitro* and after culture for several hours. The number of macrophages with different numbers of BCG-mycobacteria expressed as a percentage of the total number of infected macrophages. Data are expressed as the means ± SEM from three independent experiments. In each experiment, more than 1000 cells were analyzed at each time point.* P* < 0.05 (comparisons of the number of macrophages with 1_4 BCG-mycobacteria^&^ for the following time points (in hours): (b) 4 versus 96 and 120 and (c) 24 versus 96 and 120; with 20_50 BCG-mycobacteria^*∗*^ for the following time points (in hours): (b) 4 versus 72, 96, and 120 and (c) 24 versus 72, 96, and 120; and with >50 BCG-mycobacteria^*∗∗*^ for time points (in hours): (b) 96 versus 120 and (c) 4 versus 72, 96, and 120).

Granulomas from mouse bone marrow (BM/) and spleens (S/) were isolated on day 20 (/20 d) after infection with BCG* in vivo*, and splenic granulomas were isolated one month (/1 m) and two months (/2 m) following infection. All the granulomas were seeded into culture plates. The granulomas isolated after 20 days (this is how long it takes mice to develop adaptive immunity to BCG [9]), one month, and two months following infection were denoted as Gran/20 d, Gran/1 m, and Gran/2 m, respectively. Because none of the mice had been observed to have acute tuberculous infection at the time of granuloma isolation, it was concluded that these granulomas were isolated at the latent stage of BCG infection. Monolayer cultures of cells that had migrated from each granuloma were obtained. Any of the monolayer cultures of granuloma cells that may or may not retain cell clusters in the center of granulomatous lesions will be referred to as the “granuloma” throughout. The cellular composition of each granuloma and the relationships between BCG-mycobacteria and host cells in granulomas isolated from mice after 20 days, one month, and two months following infection had been characterized previously [11, 12]. Analysis of acid-fast BCG-mycobacterial loads in mouse granuloma cells throughout 48–120 h of* ex vivo* culture demonstrated that the mouse granuloma cells each basically contained a single BCG organism at all times following BCG infection* in vivo* and at any time point of* ex vivo* culture (Figure 1(a)).

**Figure 2:**
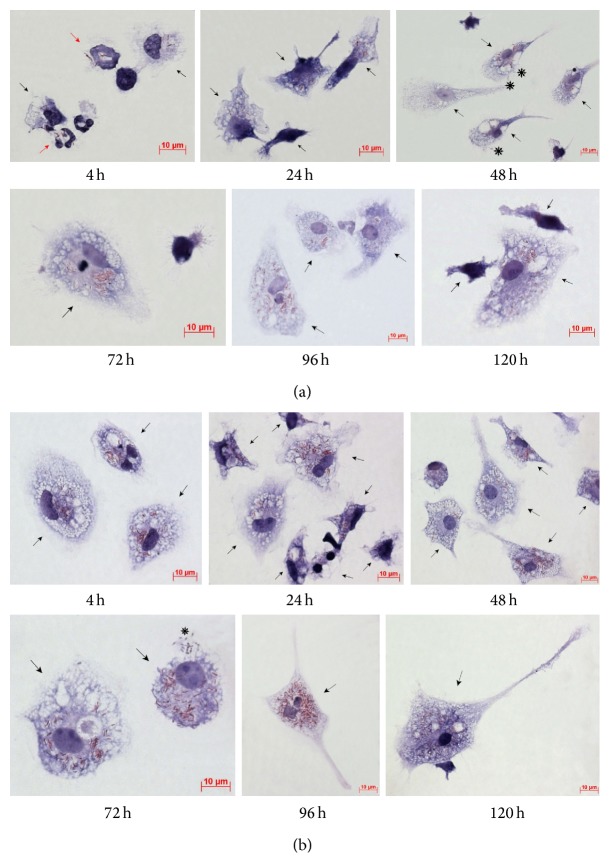
Cells with BCG-mycobacteria in the cultures of (a) bone marrow macrophages and (b) peritoneal macrophages following infection with the BCG vaccine* in vitro* and after culture for several hours. Acid-fast BCG-mycobacteria stained by the ZN method. The scale bars are 10 *μ*m each. Infected macrophages and neutrophils are indicated by the black and red arrows, respectively. Nonacid-fast BCG-mycobacteria (blue color) eliminated by macrophages are indicated by the black snowflakes. Abbreviations as in Figure 1.

The acid-fast BCG loads in the cultures of bone marrow cells and peritoneal macrophages were analyzed throughout 4–120 h following BCG infection* in vitro* (Figures 1(b)-1(c) and 2(a)-2(b)). While up to 60% of macrophage precursor cells of bone marrow each contained as few as two mycobacteria on average at hour 4 following BCG infection, we thereafter observed a decrease in the number of cells as those with a gradual increase in the occurrence of macrophages each containing more than 20 BCG-mycobacteria (Figure 1(b)). At hour 120 following infection, a considerable number of macrophages in the bone marrow cell population had increased mycobacterial loads (Figures 1(b) and 2(a)). In parallel, we observed a decrease in the number of macrophages containing a single BCG organism (Figure 1(b)). In bone marrow cell cultures, a large number of neutrophils were observed to contain acid-fast BCG-mycobacteria at hour 4 following infection (Figure 2(a)). 36.52 ± 4.76%, 15.28 ± 4.75%, and 3.37 ± 0.43% of neutrophils were observed to contain 1_4, 5_9, and 10_20 BCG-mycobacteria, respectively. From hour 24 following BCG infection on, very few neutrophils were observed in bone marrow cell cultures, and those observed had no acid-fast mycobacteria in them. Throughout 24–120 h following BCG infection, bone marrow cell populations were largely composed of macrophages with diverse numbers of mycobacteria in them, a limited number of fibroblasts, some of which contained a single BCG organism, and a few dendritic cells without BCG-mycobacteria.

In the mouse peritoneal macrophage cultures infected with BCG* in vitro,* increased acid-fast BCG-mycobacterial loads in a large number of cells were first observed at hour 4 following infection (Figures 1(c) and 2(b)). However, at hour 24 following infection, the number of cells as those was dramatically reduced, probably due to necrotic cell death, which had been indicated by the typical morphology of some cells in this population at hour 4 following infection. Throughout 48–96 h following infection, we observed a gradual increase in the number of viable macrophages with increased BCG loads in them and a decrease in the number of cells with one bacterium in each (Figures 1(c) and 2(b)). Some fibroblasts found in low numbers in the populations of peritoneal macrophages at hour 96 and 120 following infection each contained a single BCG organism. No BCG-mycobacteria were found in the dendritic cells identified in the peritoneal macrophage cultures.

Overall, our data suggest an active increase in the number of acid-fast mycobacteria in the macrophages of mouse bone marrow cell populations and mouse peritoneal macrophages within 4–120 h after BCG infection* in vitro*, while granuloma macrophages from the bone marrow and spleens of mice with latent tuberculous infection restricted BCG-mycobacterial reproduction in* ex vivo* culture.

### 3.2. Death of Macrophages with Increased BCG Loads in the Cultures of Bone Marrow Cells and Peritoneal Macrophages from Mice after BCG Infection* In Vitro*

**Figure 3:**
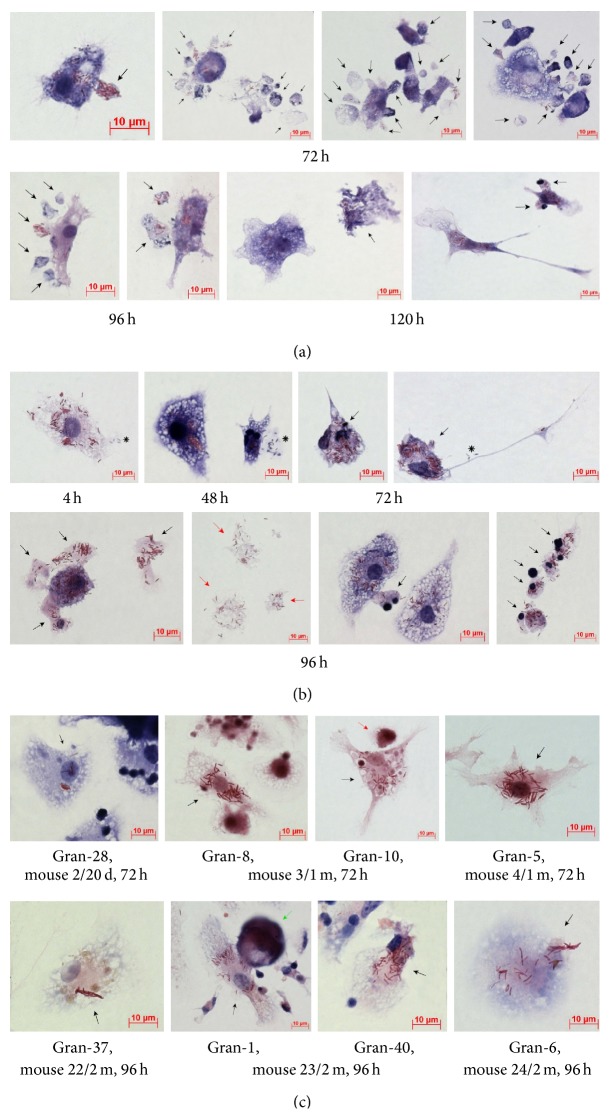
Dead (apoptotically or necrotically) and live macrophages containing an increased number of BCG-mycobacteria. Acid-fast BCG-mycobacteria stained by the ZN method. The scale bars are 10 *μ*m each. ((a)-(b)) Cells with the largest number of BCG-mycobacteria and morphological evidence of death in the cultures of mouse (a) bone marrow and (b) peritoneal macrophages following infection with the BCG vaccine* in vitro* and after culture for several hours. Apoptotic bodies and pieces of the macrophage cytoplasm with or without acid-fast BCG in them are indicated by the black arrows. Cells containing the largest number of BCG-mycobacteria and no nuclei are indicated by the red arrows. Nonacid-fast BCG-mycobacteria (blue color) expelled by macrophages into the extracellular space are indicated by the black snowflakes. (c) Macrophages with the largest number of BCG-mycobacteria in them and without morphological evidence of death are indicated by the black arrows in the fragments of mouse splenic granulomas. The red arrow indicates a dendritic cell with BCG-mycobacteria in it; the green arrow indicates a megakaryocyte. Abbreviations as in Figure 1.

**Figure 4:**
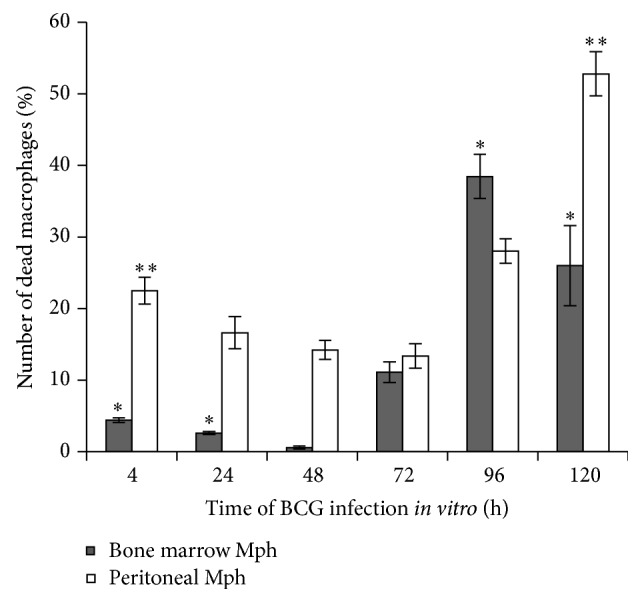
The number of dead cells is expressed as a percentage of the total number of infected macrophages (Mph) in the cultures of mouse bone marrow and peritoneal macrophages following infection with the BCG vaccine* in vitro* and after culture for several hours. Data are expressed as the means ± SEM from three independent experiments. In each experiment, more than 1000 cells were analyzed at each time point.* P* < 0.05 (comparisons of the number of dead bone marrow Mph^*∗*^ for the following time points (in hours): 4 and 24 versus 96 and 120; dead peritoneal Mph^*∗∗*^ for the following time points (in hours): 4 versus 120).

Macrophages with morphological signs of cell death (nuclei with intense chromatin condensation, nuclear fragmentation, the presence of apoptotic bodies with or without BCG-mycobacteria in them, compromised cytoplasmic membranes, leakage of cell components with or without BCG-mycobacteria in them, and the occurrence of nucleus-free cells and chromatolysis (Figures 3(a)-3(b))) were observed in the cultures of bone marrow cells and peritoneal macrophages since the onset of acute BCG infection* in vitro* (Figures 3(a)-3(b) and 4); however, none of the peritoneal or bone marrow macrophages were apoptotic or necrotic in the control (uninfected) cultures* in vitro* at any time point. The number of dead macrophages in the cultures of bone marrow cells increased considerably starting from hour 72 following infection (Figures 3(a) and 4). This result was in agreement with the increase in the number of BCG-mycobacteria in these macrophages (Figures 1(b) and 2(a)). At hour 96 following infection, the number of dead macrophages exceeded 35% of the bone marrow cell population (Figure 4). Each dead macrophage normally had more than 21 BCG-mycobacteria in it.

The cultures of peritoneal macrophages had quite a different pattern of cell death after infection with BCG* in vitro* (Figures 3(b) and 4). At hour 4 following infection, more than 20% of macrophages showed morphological evidence of being dead (Figure 3(b)). The dead cells (peritoneal macrophages) normally contained a large number of ingested BCG-mycobacteria. However, as the culture went on, there was a decrease in the number of dead macrophages: at hour 72 following infection, they made up 10% of the then current cell population at that point. However, at hour 96 following infection, dead cells made up 30% of the then current cell population (Figures 3(b) and 4), which was in correlation with the increase in the number of BCG-mycobacteria in the peritoneal macrophages (Figures 1(c) and 2(b)). At hour 120 following infection, dead macrophages made up 50% of the then current cell population (Figure 4). Each dead peritoneal macrophage contained more than one BCG organism in it—as many as each bone marrow macrophage did. Notably, some peritoneal and bone marrow macrophages with increasing mycobacterial load had the capability for phagocytosis and destruction of damaged cells, mainly lymphocytes, throughout 72–120 h following BCG infection* in vitro*. Phagosomes with engulfed lymphocytes at various stages of degradation existed side by side with large numbers of acid-fast BCG-mycobacteria in the bone marrow (Figure 2(a)) and peritoneal macrophages (Figure 2(b)).

**Figure 5:**
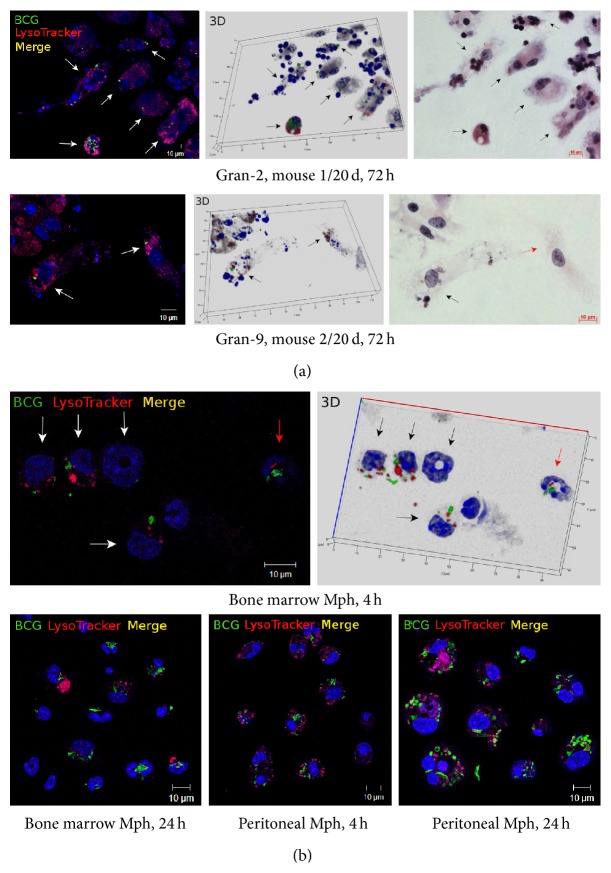
Representative confocal fluorescent images of macrophages (Mph) stained by the LysoTracker Red DND-99 dye (red signal) and mycobacterial LAM-specific antibodies (green signal) show lack of colocalization of BCG-mycobacteria with host cell lysosomes (lack of yellow signal). Nuclei are stained by DAPI (blue signal). The scale bars are 10 *μ*m each. (a) Fragments of splenic granulomas from mouse 2 on 20 days following BCG infection* in vivo*. In the right panels, the same fragments as in the other panels restained for acid-fast BCG-mycobacteria by the ZN method. Macrophages containing BCG-mycobacteria are indicated by the white arrows on the fluorescent images (left panels) and the black arrows on the 3D (central panels) and ZN (right panels) images. The red arrow in the lower-right panel indicates a macrophage, which does not contain acid-fast BCG-mycobacteria but has one LAM-labeled BCG-*Mycobacterium* in it on the fluorescent images (left and central panels). (b) Cells with BCG-mycobacteria in the cultures of mouse bone marrow and peritoneal macrophages following infection with the BCG vaccine* in vitro* and after culture for several hours. Macrophages and neutrophils with BCG-mycobacteria in them are indicated by the white and red arrows, respectively, on the fluorescent image (upper-left panel) and the black and red arrows, respectively, on the 3D image (upper-right panel). Abbreviations as in Figure 1.

In mouse granulomas at all times following BCG infection* in vivo* and of* ex vivo* culture, macrophages with large numbers of BCG-mycobacteria, including those in cording colonies [12], exhibited no morphological signs of cell death and retained their capacity for phagocytosis and disposal of damaged granuloma cells, mainly lymphocytes and platelets (Figure 3(c)). No colocalization of the acidophilic LysoTracker probe and BCG-mycobacteria detected by antibodies reacting with the major mycobacterial cell wall component glycolipid lipoarabinomannan (LAM) was observed in granuloma macrophages, whether with single or multiple BCG-mycobacteria in them (Figure 5(a)). The LAM-labeled BCG-mycobacteria that had avoided host killing in lysosomes and survived and reproduced within the granuloma macrophages were largely acid-fast, with the integrity of their cell walls preserved and, therefore, viable (Figure 5(a)). Note that no colocalization of LAM-labeled BCG-mycobacteria with the acidotropic LysoTracker dye in the macrophages or neutrophils was observed in the cultures of bone marrow cells and peritoneal macrophages throughout 4–24 h following BCG infection* in vitro* either (Figure 5(b)).

Thus, increased death rates were revealed, throughout 72–120 h following BCG infection* in vitro* in the cultures of bone marrow and peritoneal macrophages, for macrophages heavily loaded with BCG-mycobacteria, while in* ex vivo* cultures granuloma macrophages with large numbers of BCG-mycobacteria in them were still viable and had neither apoptotic nor necrotic morphology.

### 3.3. Production of IFN*γ*, IL-1*α*, and GM-CSF in Granuloma Cells from Mice with Latent Tuberculous Infection and in Bone Marrow and Peritoneal Macrophages after BCG Infection* In Vitro*

**Figure 6:**
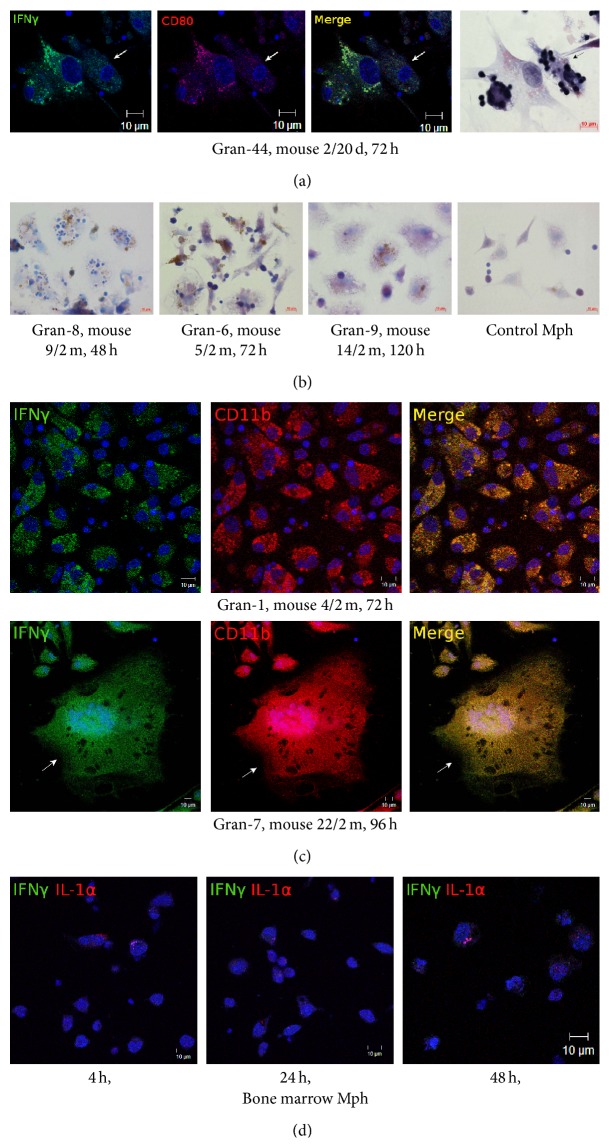
((a)–(c)) The cells with IFN*γ* in the fragments of mouse splenic granulomas. The scale bars are 10 *μ*m each. ((a), (c)) Colocalization of the markers on the confocal images of cells (yellow signal). ((a), (c)-(d)) Nuclei stained by DAPI (blue signal). (a) Confocal immunofluorescent localization of IFN*γ* (green signal) and CD80 (red signal) in granuloma macrophages (Mph) and a fibroblast at the center of the images. In the right panel, the same fragment as in the other panels restained for acid-fast BCG-mycobacteria by the ZN method. An IFN*γ*-producing macrophage with BCG-mycobacteria reproducing in it is indicated by the white arrows on the fluorescent images and the black arrow on the ZN image. (b) Immunochemical localization of IFN*γ* in granuloma cells and control mouse peritoneal macrophages. The brown color indicates the presence of IFN*γ* in these cells. (c) Confocal immunofluorescent localization of IFN*γ* (green signal) and CD11b (red signal) in granuloma macrophages, with a multinucleate Langhans giant cell indicated by the white arrows in the three lower panels. (d) Bone marrow macrophages following infection with the BCG vaccine* in vitro* and after culture for several hours not stained by the IFN*γ*- (green signal) and IL-1*α*-specific antibodies (red signal) on the confocal fluorescent images. Abbreviations as in Figure 1.

**Figure 7:**
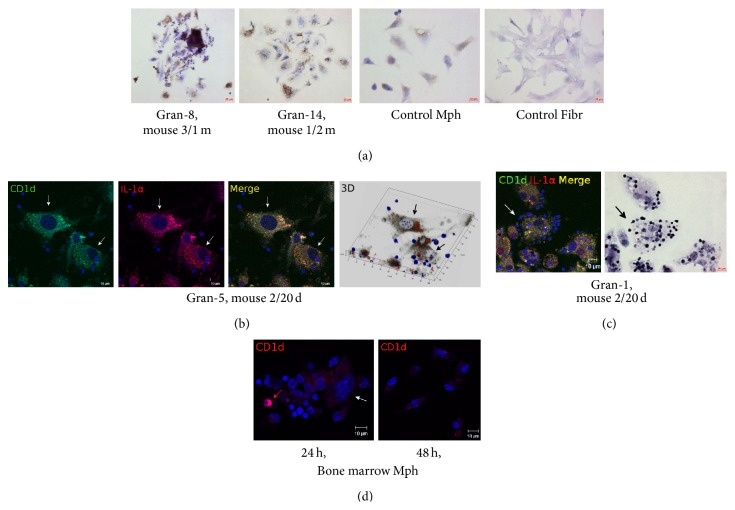
((a)–(c)) Bacterial lipid- and glycolipid-presenting molecule CD1d in the cells in the fragments of mouse splenic granulomas. The scale bars are ((a, central right panel), (b)–(d)) 10 *μ*m each and (a, other panels) 20 *μ*m each. ((b)-(c)) Colocalization of the markers on the confocal images of cells (yellow signal). ((b)–(d)) Nuclei stained by DAPI (blue signal). (a) Immunochemical localization of CD1d in granuloma cells, control mouse peritoneal macrophages (Mph), and fibroblasts (Fibr). The brown color indicates the presence of CD1d in these cells. ((b)-(c)) Confocal immunofluorescent localization of CD1d (green signal) and IL-1*α* (red signal) in granuloma macrophages and fibroblasts. (b) CD1d- and IL-1*α*-producing fibroblasts are indicated by the white arrows on the fluorescent images and the black arrows on the 3D image. (c) In the right panel, the same fragment as in the left panel restained for acid-fast BCG-mycobacteria by the ZN method. A CD1d- and IL-1*α*-producing macrophage with BCG-mycobacteria reproducing in it is indicated by the white arrow on the fluorescent image and the black arrow on the ZN image. (d) Confocal immunofluorescent localization of CD1d (red signal) in the cells in the cultures of bone marrow macrophages following infection with the BCG vaccine* in vitro* and after culture for several hours. The macrophage with CD1d in it is indicated by the red arrow, and a megakaryocyte without the antigen is indicated by the white arrow. Abbreviations as in Figure 1.

It is well established that the cytokines IFN*γ*, IL-1, and GM-CSF are inflammatory mediators, which activate and differentiate immune cells, increasing their microbicidal potential in response to infection with intracellular pathogens and thus playing an important role in initiating/regulating inflammation and controlling infection [7, 13, 18, 26, 27]. An immunocytochemical and an immunofluorescence assay of IFN*γ*, cell-associated IL-1*α*, and GM-CSF demonstrated their active production in granuloma cells (macrophages, multinucleate Langhans giant cells, and fibroblasts) from the spleens of mice after 20 days, one month, and two months following infection with the BCG vaccine* in vivo* and after* ex vivo* culture for 48–96 h (see Table 1, Figures 6(a)-6(b), 7(b)-7(c) and 10(c)). Noteworthy, cytokine-producing granuloma macrophages with increased microbicidal potential contained replicating acid-fast BCG-mycobacteria too (Figures 6(a), 7(c), and 10(c)). However, neither peritoneal nor bone marrow macrophages—whether after BCG infection* in vitro* or in the control group—were observed to synthesize these cytokines (see Table 1, Figures 6(b)-6(c)). Interestingly, there was one-fifth as many IFN*γ*-synthesizing cells in the mouse 14/2 m granulomas assayed at hour 120 of* ex vivo* culture (see Table 1, Figure 6(b)) as there was in the mouse granulomas assayed throughout 48–96 h of* ex vivo* culture. There was no difference in BCG load in granuloma macrophages or dendritic cells [12] between mouse 14/2 m and any of mice 3/2 m, 4/2 m, 5/2 m, 9/2 m, and 22/2 m assayed for IFN*γ* production throughout 48–96 h of* ex vivo* culture. To answer the question as to whether this considerable reduction in the number of IFN*γ*-producing cells in mouse 14/2 m granulomas was due to the longer time of* ex vivo* culture or some individual features (whatever they are) of mouse 14/2 m were factors, further research is required.

Thus, a considerable production of proinflammatory cytokines has been observed in the mouse granuloma macrophages that largely had a single BCG organism in each of them, while peritoneal and bone marrow macrophages with high bacterial loads following BCG infection* in vitro* have not been observed to induce the synthesis of such cytokines.

### 3.4. Сell Surface Markers in Splenic Granuloma Cells from Mice with Latent Tuberculous Infection and in Bone Marrow Macrophages after BCG Infection* In Vitro*

The bacterial lipid- and the glycolipid-presenting molecule CD1d participates in T-cell development, NKT-cell activation, and regulation of immunity in response to invasion by mycobacteria [27–29]. CD1d molecules were found on the surface of most macrophages (both with and without acid-fast BCG-mycobacteria in them), dendritic cells, and fibroblasts within the granulomas examined (Figures 7(a)–7(c) and 11). Peritoneal macrophages in the control groups stained most weakly for CD1d. Neither control fibroblasts obtained from the intact mice, nor the peritoneal macrophages from mice after 20 days following intraperitoneal infection with the BCG vaccine, nor the bone marrow macrophages from mice after 24–48 h following BCG infection* in vitro* stained for CD1d (Figures 7(a), 7(d), and 11). Few granuloma lymphocytes (11.13 ± 0.1% and 9.27 ± 1.14% in the granulomas from the spleens of the mice after one month and two months following infection with the BCG vaccine* in vivo*, resp.) stained for CD1d (Figure 7(a)).

**Figure 8:**
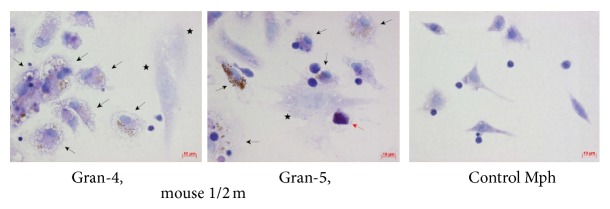
Immunochemical localization of the activation marker CD25 on the cells in the fragments of mouse splenic granulomas and control mouse peritoneal macrophages (Mph). The scale bars are 10 *μ*m each. The brown color indicates the presence of the receptor in these cells. Macrophages and dendritic cells with CD25 in them are indicated by the black and red arrows, respectively. The black stars indicate the fibroblasts that do not contain the antigen. Abbreviations as in Figure 1.

СD25 is a marker of cell activation and the *α* chain of the receptor for IL-2 participating in the development of the inflammatory response to alien antigens and activating antigen-presenting functions of cells of the immune system. IL-2R*α* can combine with the other two chains of the receptor to form high-affinity signaling receptor complexes for IL-2 when macrophages and T cells are activated [30]. Additionally, СD25 is one of the markers of regulatory T cells [30]. The antibodies reacted with CD25 in 20–25% of macrophages, 4-5% of dendritic cells, and 6–12% of lymphocytes in the mouse granulomas (Figures 8 and 11). Note that the number of lymphocytes containing СD25 was on average higher in granulomas from the mice after two months than one month following BCG infection* in vivo* (Figure 11). The granuloma fibroblasts did not stain for this marker (Figure 8). CD25 was not found in peritoneal macrophages from the control mice, peritoneal macrophages from mice after 20 days following intraperitoneal infection with the BCG vaccine, or bone marrow macrophages at hour 48 following BCG infection* in vitro* (Figures 8 and 11).

**Figure 9:**
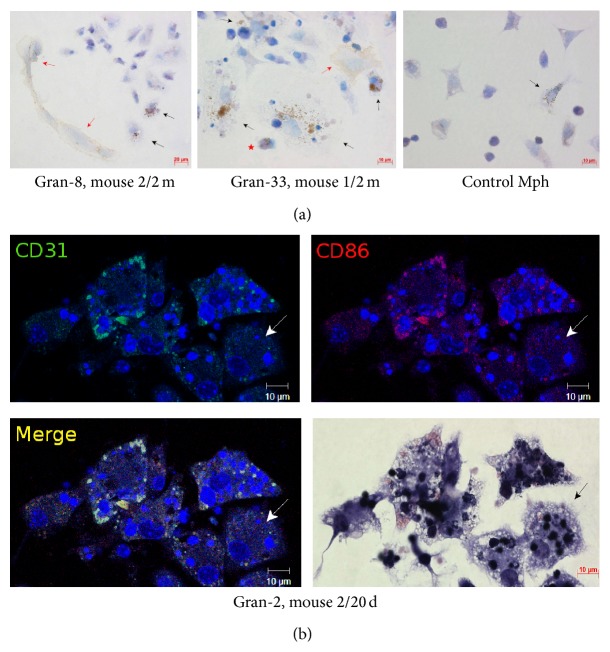
The cell-to-cell adhesive molecule CD31 on the cells in the fragments of mouse splenic granulomas. The scale bars are (a, left panel) 20 *μ*m and ((a, other panels)-(b)) 10 *μ*m each. (a) Immunochemical localization of CD31 on granuloma cells and control mouse peritoneal macrophages (Mph). The brown color indicates the presence of the receptor in these cells. The black arrows, red arrows, and red stars indicate macrophages, fibroblasts, and dendritic cells, respectively, stained for this marker. (b) Confocal immunofluorescent localization of the cell-surface receptor CD31 (green signal) and the costimulatory molecule CD86 (red signal) on granuloma macrophages. Colocalization of the markers on confocal images of cells (yellow signal). Nuclei are stained by DAPI (blue signal). In the lower-right panel, the same fragment as in the other panels restained for acid-fast BCG-mycobacteria by the ZN method. A CD31-producing macrophage with BCG-mycobacteria reproducing in it is indicated by the white arrows on the fluorescent images and by the black arrow on the ZN image. Abbreviations as in Figure 1.

CD31, also known as PECAM-1 (Platelet Endothelial Cell Adhesion Molecule 1), is a transmembrane glycoprotein, one of the adhesion molecules in the immunoglobulin superfamily. Its primary function is the mediation of leukocyte-endothelial cell adhesion and signal transduction in various inflammatory disorders [31]. CD31 was found on the surface of 17–27% of macrophages, both with and without acid-fast BCG-mycobacteria in them, and on the surface of 30–40% of dendritic cells in granulomas from all mice infected with the BCG vaccine* in vivo* (Figures 9 and 11). CD31 was not detected on fibroblasts from the spleens of the control mice, nor was it found on mouse granuloma fibroblasts (Figure 9(a)). The peritoneal macrophages obtained from the intact mice or the mice after 20 days following intraperitoneal infection with the BCG vaccine stained little for CD31 (Figures 9 and 11).

**Figure 10:**
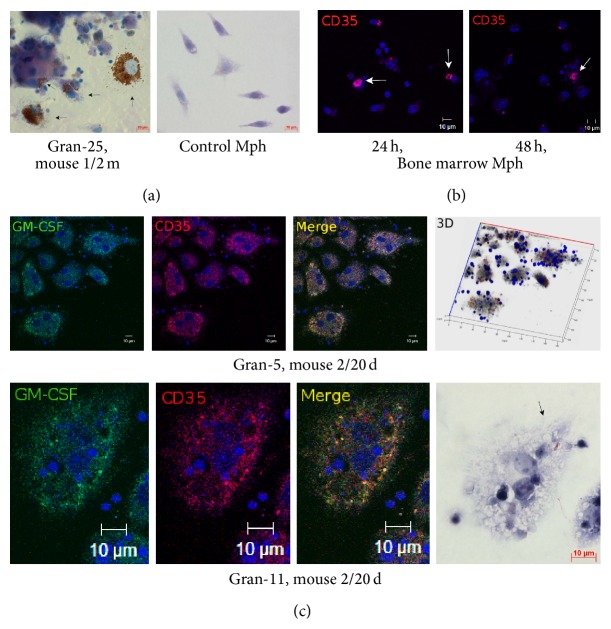
The phagocytic receptor CD35 on the cells in the fragments of mouse splenic granulomas. The scale bars are 10 *μ*m each. ((b)-(c)) Nuclei stained by DAPI (blue signal). (a) Immunochemical localization of CD35 on granuloma cells and control mouse peritoneal macrophages (Mph). The brown color indicates the presence of the receptor in these cells. The black arrows indicate macrophages stained for this marker. (b) Confocal immunofluorescent localization of CD35 (red signal) on the cells in the cultures of bone marrow macrophages following infection with BCG* in vitro* and after culture for several hours. Macrophages stained for CD35 are indicated by the white arrows. (c) Confocal immunofluorescent localization of GM-CSF (green signal) and CD35 (red signal) in granuloma macrophages. In the lower-right panel, the same fragment as in the other panels restained for acid-fast BCG-mycobacteria by the ZN method. The black arrow on the ZN image indicates the macrophage region with BCG-mycobacteria reproducing in it. Abbreviations as in Figure 1.

**Figure 11:**
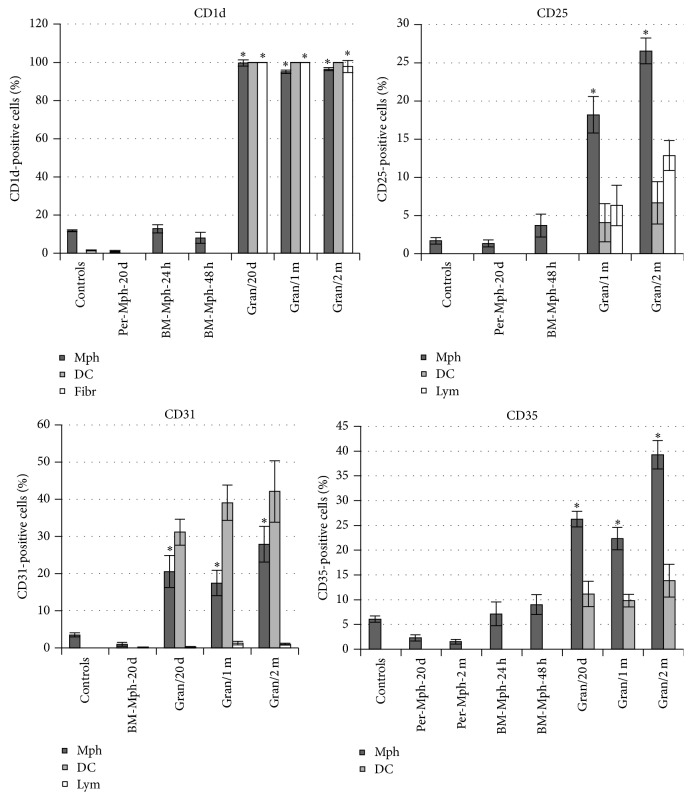
Macrophages (Mph), dendritic cells (DC), fibroblasts (Fibr), and lymphocytes (Lym) with cell-surface markers CD1d, CD25, CD31, and CD35. The objects examined are control peritoneal macrophages and splenic fibroblasts from three intact mice, peritoneal and bone marrow macrophages obtained from two mice on day 20 following intraperitoneal infection with the BCG vaccine (Per-Mph-20 d and BM-Mph-20 d, resp.), peritoneal macrophages obtained from mouse 25 after two months following intraperitoneal infection with the BCG vaccine (Per-Mph-2 m), mouse bone marrow macrophages following infection with the BCG vaccine* in vitro* and after culture for several hours (BM-Mph-24 h and BM-Mph-48 h), and granulomas (Gran) from the spleens of two to four mice after 20 days (Gran/20 d), one month (Gran/1 m), and two months (Gran/2 m) following BCG infection* in vivo* and* ex vivo* culture for several hours. Granuloma cells producing various surface antigens were analyzed in each granuloma separately; the values were averaged for all the mice and then combined for granulomas in each group. In each experiment, more than 1000 cells were analyzed at each time point. Data are expressed as the means ± SEM. ^*∗*^ *P* < 0.01 (comparisons of the number of CD-positive macrophages in each Gran group and controls, BM-Mph-24 h, BM-Mph-48 h, Per-Mph-20 d, BM-Mph-20 d, and Per-Mph-2 m).

CD35, also known as the complement receptor type 1 (CR1), participates in phagocytosis of С3b-opsonized mycobacteria and promotes microbial death in the phagosomes of host cells [32, 33]. CR1 was found on the surface of 22–39% of the macrophages, both with and without acid-fast BCG-mycobacteria, and 9–13% of the dendritic cells in mouse granulomas (Figures 10(a), 10(c), and 11). The peritoneal macrophages obtained from the intact mice or the mice after 20 days and after two months following intraperitoneal infection with the BCG vaccine did not stain for CD35 (Figures 10(a) and 11). CD35 was not detected on fibroblasts from the spleens of the control mice or mouse granulomas. Also, bone marrow macrophages stained little for CD35 throughout 24–48 h after BCG infection* in vitro* (Figures 10(c) and 11).

Thus, this study of the cell-surface markers demonstrates significant activation of granuloma cells through the expression of various receptors in the mouse granulomas with low mycobacterial loads; however, bone marrow macrophages that had been infected with BCG* in vitro* and had very high bacterial loads were not observed to produce any of those activation-related molecules.

### 3.5. Extracellular Matrix and Cytoskeletal Proteins in Splenic Granuloma Cells from Mice with Latent Tuberculous Infection

**Figure 12:**
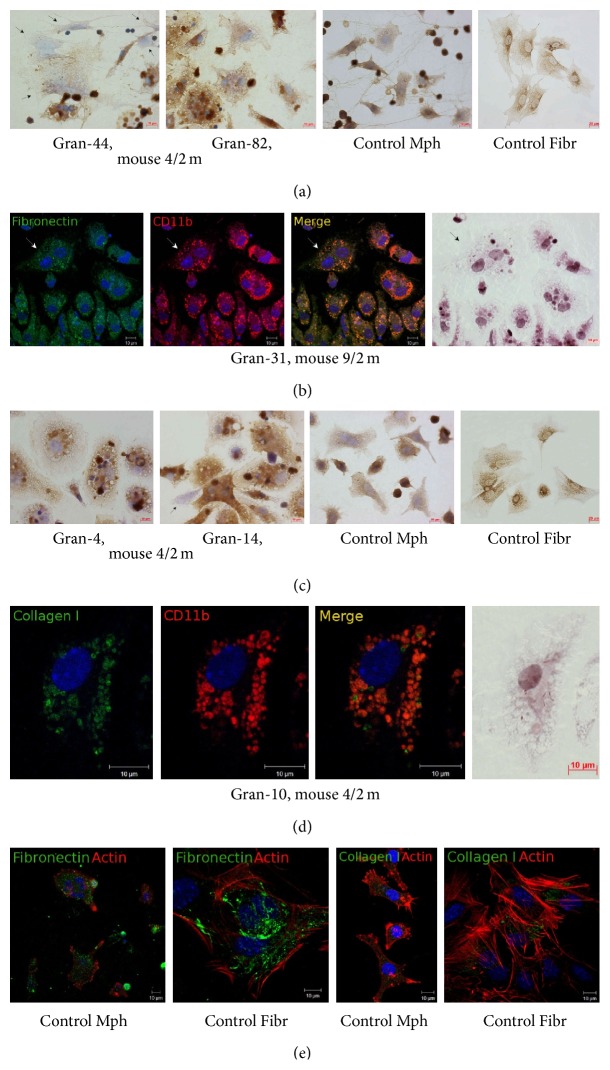
Fibronectin and type I collagen (collagen I) in the cells in the fragments of mouse splenic granulomas. The scale bars are ((a, right panel), (c, right panel)) 20 *μ*m each and ((a)–(e)) 10 *μ*m each. ((b), (d)-(e)) Colocalization of the markers on confocal images of cells (yellow signal). Nuclei stained by DAPI (blue signal). ((a), (c)) Immunochemical localization of (a) fibronectin and (c) type I collagen in granuloma cells and control mouse peritoneal macrophages (Mph) and splenic fibroblasts (Fibr). The brown color indicates the presence of the markers in these cells. The black arrows indicate fibroblasts; the other cells are macrophages, dendritic cells, and lymphocytes. (b) Confocal immunofluorescent localization of fibronectin (green signal) and CD11b (red signal) in granuloma macrophages. In the right panel, the same fragment as in the other panels restained for acid-fast BCG-mycobacteria by the ZN method. A macrophage with BCG-mycobacteria reproducing in it is indicated by the white arrows on the fluorescent images and the black arrow on the ZN image. (d) Confocal immunofluorescent localization of type I collagen (green signal) and CD11b (red signal) in a granuloma macrophage with BCG-mycobacteria. In the right panel, the same fragment as in the other panels restained for acid-fast BCG-mycobacteria by the ZN method. (e) Confocal immunofluorescent localization of fibronectin (green signal), type I collagen (green signal), and filamentous actin (red signal) in the control peritoneal macrophages and fibroblasts. Abbreviations as in Figure 1.

**Figure 13:**
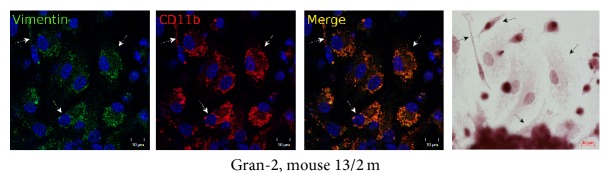
Confocal immunofluorescent localization of vimentin (green signal) and CD11b (red signal) in the cells in a fragment of a mouse splenic granuloma. The scale bars are 10 *μ*m each. Colocalization of the markers on the confocal images of cells (yellow signal). Nuclei are stained by DAPI (blue signal). In the right panel, the same fragment as in the other panels restained for acid-fast BCG-mycobacteria by the ZN method. Macrophages with BCG-mycobacteria reproducing in them are indicated by the white arrows on the fluorescent images and the black arrows on the ZN image. Abbreviations as in Figure 1.

It has been established that mycobacteria have evolved a wide range of molecules, known as adhesions, to enable binding with fibronectin, a protein of the host extracellular matrix, and type I collagen fibers and, eventually, to invade host cells [34]. The cytoskeletal filamentous proteins actin and vimentin are responsible for cell migration, attachment to the substrate, maintaining cell shape, integrity of the cytoplasm, phagocytosis of bacterial agents, and elimination of intracellular pathogens [34]. According to preliminary results of the immunocytochemical and the immunofluorescence assay, the production rates for fibronectin, type I collagen, filamentous actin, and vimentin in granuloma macrophages were not affected by mycobacterial loads, nor were those in granuloma fibroblasts from mice following infection with BCG vaccine* in vivo* or in peritoneal macrophages and splenic fibroblasts from the control group of intact mice (Figures 12(a)–12(e) and 13). No colocalization of BCG-mycobacteria with filamentous actin in granuloma macrophages was observed either (data not shown).

**Figure 14:**
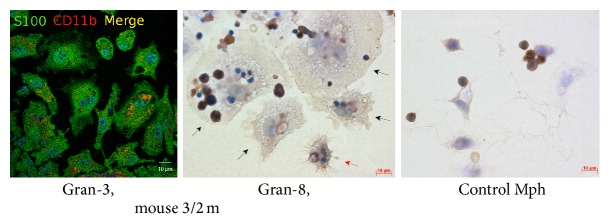
The S100 proteins in the cells in the fragments of mouse splenic granulomas and control mouse peritoneal macrophages (Mph). The scale bars are 10 *μ*m each. In the left panel, confocal immunofluorescent localization of S100 proteins (green signal) and CD11b (red signal). Colocalization of the markers on confocal images of cells (yellow signal). Nuclei are stained by DAPI (blue signal). In the central and right panels, immunochemical localization of S100 proteins in the cells. The brown color indicates the presence of the markers in these cells. The black and red arrows indicate macrophages and a dendritic cell, respectively, with increased production of S100 proteins. Abbreviations as in Figure 1.

The S100 proteins are a large family of cytoplasmic and extracellular Ca^2+^-binding molecules regulating enzyme activities, the dynamics of cytoskeletal constituents, Ca^2+^ homeostasis, and the inflammatory response [35]. The S100 proteins regulate intermediate filaments, microfilaments and other cytoskeletal proteins, cell morphology and motility, and the reciprocal relationships of cytoskeletal elements [36]. Mouse granuloma macrophages stained much stronger with polyclonal antibodies reactive with the S100 proteins than peritoneal macrophages from the control mice (Figure 14).

Thus, a high expression of cytokines and other activation-related CD markers, the unchanged synthesis of constitutive cytoskeletal and extracellular matrix proteins, was observed in granuloma cells with low mycobacterial loads from mice with latent tuberculous infection. By contrast, no expression of the cytokines or CD markers was detected in the control cultures of uninfected cells or peritoneal and bone marrow macrophages with high bacterial loads following acute BCG infection* in vitro*.

## 4. Discussion

In the present study, we have for the first time compared* Mycobacterium*-host cell relationships in individual granuloma cells from the bone marrow and spleens of mice with latent tuberculous infection and in the cells from mouse bone marrow and peritoneal cultures after BCG infection* in vitro.* The study of BCG loads in mouse granuloma macrophages at the latent stage of tuberculous infection demonstrated that, although there was individual variation in the number of bacteria across the cells of granulomas from different mice [12], those cells each normally contained a single BCG organism throughout 48–120 h of* ex vivo* culture. By contrast, a considerable increase in the number of BCG-mycobacteria was observed in the cultures of bone marrow cells and peritoneal macrophages infected with BCG* in vitro* throughout 4–120 h following infection. Therefore, mouse granuloma macrophages could control mycobacterial reproduction in cells, while the intact mice's macrophages infected* in vitro* could not do as much. In a study by Jordao and the coworkers [21], BCG-mycobacteria were killed very efficiently by mouse bone marrow-derived macrophages, but not by human monocyte-derived macrophages, in which BCG-mycobacteria actively replicated for 2–7 days after BCG infection* in vitro*.

As is known, the proinflammatory cytokines IFN*γ*, TNF*α*, and IL-1 synthesized by macrophages, lymphocytes, and other cells of the immune system in animals control tuberculous infection [37–39]. It has been demonstrated that the action of IFN*γ* on cells infected with mycobacteria* in vitro* leads to activation of inducible NO synthase and necrotic/apoptotic death of infected cells [21, 26, 40]. In our work, analysis of the production of the proinflammatory cytokines IFN*γ*, IL-1*α*, and GM-CSF demonstrated significant activation of their synthesis in mouse granuloma cells after 20 days, one month, and two months following infection with the BCG vaccine* in vivo* and at different times of* ex vivo* culture, while mouse bone marrow cells and peritoneal macrophages infected with the BCG vaccine* in vitro* were not observed to induce their synthesis. As a result, we observed a considerable increase in the number of BCG-mycobacteria in acutely infected bone marrow and peritoneal macrophages in mice, causing increased death rates for host cells. In contrast to the cultures of cells infected with the BCG vaccine* in vitro*, mouse granuloma macrophages were not observed to have a burst-like increase in BCG-mycobacterial population during* ex vivo* culture or to die because of increased BCG-mycobacterial loads. Our results are not consistent with those reported by Lee and Kornfeld [40], who had added recombinant cytokine IFN*γ* to the growth medium with mouse bone marrow macrophages infected with BCG-mycobacteria and* M. tuberculosis in vitro* and observed increased death rates for the cells that were heavily loaded with mycobacteria. However, our results are consistent with the conclusion made by the same authors [40] that IFN*γ* promotes survival of macrophages with a low intracellular bacterial load in them by inhibiting mycobacterial reproduction. Zhang and the coworkers [41] demonstrated that one of the mechanisms that contribute to the restriction of* M. tuberculosis* reproduction in host cells could be depletion of tryptophan in activated macrophages through IFN*γ*-mediated upregulation of expression of the host enzyme that degrades tryptophan in mouse cells, which reduces the amount of tryptophan required for intracellular mycobacteria to reproduce actively.

Curiously, we revealed that host cells containing large numbers of BCG-mycobacteria in the bone marrow cell cultures and peritoneal macrophage cultures had been dying out at increased rates without whatever external factor, not with, for example, recombinant IFN*γ* used by Lee and Kornfeld [40] or Jordao and the coworkers [21]. In our study, the observed pattern of the death of peritoneal macrophages infected with BCG-mycobacteria* in vitro* demonstrated that macrophages are able to ingest large enough numbers of mycobacteria; however, some contained too many to deal with, and so they died in the first hours following infection. The remaining macrophages, which had ingested few mycobacteria, were viable until the growing bacterial populations reached the size at which host cells could not carry on, which, again, resulted in more dead macrophages. A similar pattern of macrophage death was observed in BCG-infected bone marrow cell cultures with the difference that the infected bone marrow macrophage precursor cells each normally ingested no more than two BCG-mycobacteria, which had no adverse effect on their viability* in vitro*. The subsequent active reproduction of BCG-mycobacteria in the bone marrow macrophages differentiated from the precursor cells resulted in above-critical BCG-mycobacterial loads in host cells, and so those host cells died within 96–120 h of culture following BCG infection* in vitro*. Lee and the coworkers [42] found no morphological or biochemical evidence of the apoptotic death of host cells following* in vitro* infection of mouse bone marrow cells with BCG and* M. tuberculosis*. We, by contrast, observed dead BCG-infected peritoneal and bone marrow macrophages with nuclear chromatin condensation, nuclear fragmentation, and the formation of apoptotic vesicles with and without BCG-mycobacteria, which was indicative of apoptosis. Some cells had plasma membrane disruptions and no nuclei in them, which was indicative of necrosis; however, acid-fast BCG-mycobacteria were normally located in the pieces of the cytoplasm of dead cells, just as they were in the previously studied mouse granulomas [12], in which BCG-mycobacteria had occasionally been found in apoptotic bodies and in the fragments of the cell cytoplasm located in the phagosomes of macrophages or outside cells. The apoptotic death of invaded cells is perhaps the best option for the host organism, because this leads to the elimination of the pathogen, as apoptotic bodies are ingested by nearby macrophages, and prevents the extracellular spread of mycobacteria and the development of inflammatory responses in the surrounding tissues [37, 43–45].

In this work, we compared the production rates for cytokines and activation-related CD markers in granuloma macrophages from mouse spleens at different time points of latent tuberculous infection following infection with the BCG vaccine* in vivo* and in mouse bone marrow and peritoneal macrophages following infection with the BCG vaccine* in vitro* and after culture for several hours. As a result, we revealed a considerable production of proinflammatory cytokines in the mouse granuloma macrophages that largely had a single BCG organism in each of them, while peritoneal and bone marrow macrophages with high bacterial loads following BCG infection* in vitro* were not observed to induce the synthesis of such cytokines. Note that literature data on the induction of expression of proinflammatory cytokines in animal and human cells infected with pathogenic, nonpathogenic, and attenuated mycobacterial strains* in vitro* are quite controversial. For example, Beltan and the coworkers [46] revealed a considerable induction of the synthesis of the proinflammatory cytokines IL-1, IL-6, GM-CSF, and TNF*α* after* in vitro* infection of human macrophages with only nonpathogenic mycobacteria. Aldwell and the coworkers [13], by contrast, observed enhanced expression of genes for the proinflammatory cytokines IL-1*β*, IL-6, and TNF*α* only after* in vitro* infection of bovine macrophages in bronchoalveolar fluid with pathogenic* M. bovis*, but not with attenuated BCG-mycobacteria. Similarly,* in vitro* infection of mouse bone marrow cells with highly pathogenic* M. tuberculosis *Beijing led to a stronger induction of expression of genes for the proinflammatory cytokines IL-1*β*, IL-12, and TNF*α* than did infection of cells with less virulent mycobacterial strains [47]. In a work by Rajaram and the coworkers [48], human monocyte-derived macrophages infected* in vitro* with virulent* M. tuberculosis* H37Rv and attenuated BCG-mycobacteria followed a similar pattern of activation of the production of the proinflammatory cytokine IL-8. However, in a work by Brooks and the coworkers [49], BCG-mycobacteria induced a stronger activation of the synthesis of the proinflammatory cytokine IL-1*β* in mouse bone marrow macrophages than in cells of the same type infected with* M. tuberculosis* H37Rv* in vitro*. As a result, these researchers observed a stronger reproduction of the mycobacteria of the virulent strain than BCG-mycobacteria throughout 24–168 h following infection [49]. Interestingly, the* in vitro* infection of mammalian cells with BCG-mycobacteria led to a rapid elimination of BCG-mycobacteria in the phagosomes of infected cells in some cases [15], while in others a considerable increase in the number of BCG-mycobacteria was observed during* in vitro* culture of infected cells [26, 49]. It should be noted that Sanarico and the coworkers [50] also described the BCG strain-specific modulation of the bacterium-host cell interactions in human monocyte-derived dendritic cells after acute infection with BCG Japan or BCG Aventis Pasteur in* in vitro* culture. As is known [51], BCG Japan belongs to the “early strains” of BCG as does BCG Russia used in our study. On the other hand, BCG Japan was incapable of intracellular replication, while BCG Aventis Pasteur belonging to the “later strains” enlarged the bacterial population in dendritic cells [50]. Nevertheless, the differences in the gene profile were not observed in dendritic cells infected with various BCG strains, when the highest levels of mRNA for the proinflammatory cytokines IL-1*α*, IL-1*β*, IL-6, IL-12, and TNF*α* were detected. Analysis of IFN*γ* gene expression did not show any significant modulation of the production of this cytokine in dendritic cells after BCG infection [50]. In our study, relationships between mycobacteria of the vaccine strain BCG and host cells were quite different in splenic granulomas from mice with latent tuberculous infection and in mouse cells acutely infected with BCG-mycobacteria* in vitro*. We observed, as Brooks and the coworkers did [49], a stronger reproduction of BCG-mycobacteria in the macrophages of those cultures, whose cells were not producing proinflammatory cytokines or CD markers of cell activation. Thus, our study demonstrated that the* in vitro* infection of mouse cells in culture and the* in vivo* infection of animals cause macrophages to produce different responses and entail different relationships between host cells and mycobacteria. The reasons for the inconsistent results and large variation in the intracellular growth rate of mycobacteria among different research groups are unclear; however, as reviewed by Rivero-Lezcano [52], mycobacterial strains, cell models, infection protocols, assays of mycobacterial growth, and cytokines for activation of host cells were different and so were the experimental settings. In addition, the observed differences should be taken into account whenever experimental cell models are used for testing and validation of antimycobacterial drugs and vaccines. Overall, although the differences in* Mycobacterium*-host cell relationships are actively studied by many researchers, more information is required for a better understanding of pathogen-host cell interactions. Therefore, as reviewed in [52], the development of more complex cell models that can involve most types of immune cells and many humoral immunological factors may help discover new antimycobacterial mechanisms. Consequently, we have developed a new* ex vivo* granuloma cell model from mice with latent tuberculous infection that is very similar to* in vivo* conditions, both for research into* Mycobacterium*-host cell relationships in the short term and for testing new vaccines and antimycobacterial drugs in a long term.

We have observed induction of expression of the activation markers CD1d, CD25, CD31, and CD35 on mouse granuloma macrophages at different time points of latent tuberculous infection, and not on bone marrow macrophages after infection with the BCG vaccine* in vitro*. As is known [29, 53], CD1d is an antigen-presenting receptor of nonprotein, lipid, and glycolipid molecules to a variety of T cells. LAM has been identified as a CD1 antigen in mycobacteria [29]. It is possible that some CD1d-positive mouse granuloma lymphocytes were the so-called invariant natural killer T cells, which respond very rapidly to mycobacterial infection [29]. Activation of CD25 on mouse granuloma macrophages demonstrated an increased responsiveness of the macrophages to the powerful immunoregulatory lymphokine IL-2, which stimulates macrophages and other cells of the immune system for the treatment of infectious diseases [30]. It is possible that the lymphocytes with CD25 in mouse granulomas are in the class of regulatory T cells involved in suppression of activation of the microbicidal functions of macrophages and dendritic cells with the development of immune tolerance [54], so much so that CD25-positive lymphocytes were observed to increase in number as chronic tuberculous infection in mice was progressing. The increased number of CD31 and CD35 receptors on granuloma macrophages was indicative of activation of the proinflammatory response of cells to mycobacterial infection in mice. Elevated S100 protein concentrations in mouse granuloma cells were probably due to activation of the production of сalgranulins, because these three proteins, S100A8, S100A9, and S100A12, are inducible in macrophages and are largely expressed by activated macrophages as important mediators of inflammation and microbial infections, suggesting a key role for these proteins in the innate immune response [36]. Nevertheless, although the cells of the immune system in the mice were activated, BCG-mycobacterial reproduction in mouse granuloma macrophages was observed to go on.

As is known [34], mycobacteria possess a large number of bacterial proteins that operate as bacterial adhesions for binding with proteins of the extracellular matrix, followed by infection of host cells through activation of phagocytosis-related processes. Preliminary results suggested that there was no difference in the production of the extracellular matrix proteins fibronectin and type I collagen between mouse granuloma cells, whether containing BCG-mycobacteria or not, and the control mouse macrophages. Furthermore, no differences were observed in the expression of the cytoskeletal proteins vimentin and filamentous actin. Studies of the proteins that are involved in cell-to-cell interactions and phagocytosis of bacteria in the cells operating within inflammatory foci such as granulomas will go on, because they are important for understanding the mechanisms underlying mycobacterial persistence in host cells in the settings of latent tuberculous infection in animals and man.

Consequently, the search for factors that influence the reproduction and survival of mycobacteria, including vaccine strains, in host cells and the study of relationships between cells and their intracellular pathogens, both following acute infection in* in vitro* culture and in foci of latent tuberculous infection, are the priority in investigating the role of the cells of the immune system in the protection of man and animals against mycobacterial infections caused by pathogenic or nonpathogenic mycobacteria.

## 5. Conclusions

BCG has been used as a vaccine for immunization of many people worldwide; however, the results are varying. Considering the situation, research into the host cell response to BCG-mycobacteria during acute and latent chronic infection of animal cells is very important for developing a more rational approach to the improvement of the BCG vaccine. In the present study, we compared* Mycobacterium*-host cell relationships in individual granuloma cells from mice with latent tuberculous infection and cells from mouse bone marrow and peritoneal cultures infected with BCG* in vitro*. This comparison showed different specific cellular responses to latent chronic and acute BCG infection* in vitro.* We have demonstrated that mouse granuloma macrophages with an increased production of proinflammatory cytokines, growth factors, and CD receptors for cell activation cannot eliminate intracellular BCG-mycobacteria. Nevertheless, these macrophages can control mycobacterial reproduction in host cells both* in vivo* and in* ex vivo* culture, while those not activated after acute BCG infection* in vitro* cannot. We expect that our results will add to the understanding of BCG-*Mycobacterium*-host cell interactions and may facilitate the design and improvement of vaccines against tuberculosis.

## Figures and Tables

**Table 1:** Proinflammatory cytokines IFN*γ* and IL-1*α* and growth factor GM-CSF in the macrophages of granulomas (Gran) isolated from the spleens of *n* mice after 20 days (/20 d), one month (/1 m), and two months (/2 m) following BCG infection *in vivo* and after *ex vivo *culture, in peritoneal (Per-Mph) and bone marrow macrophages (BM-Mph) infected with BCG *in vitro* (BCG) or without infection (controls), in the peritoneal macrophages isolated from mice on day 20 after intraperitoneal BCG infection (Per-Mph-20 d), and after culture for several hours.

Cells	Cytokine-positive cells, %			
	IFN*γ*		IL-1*α*	GM-CSF
	Macrophages	Lymphocytes	Macrophages	Macrophages
Per-Mph-control-24 h	0.51 ± 0.41	0	2.91 ± 1.45	3.75 ± 0.56
Per-Mph-BCG-4 h	0.28 ± 0.2	0	7.07 ± 2.28	ND
Per-Mph-BCG-24 h	0.36 ± 0.23	0	3.06 ± 2.1	ND
BM-Mph-control-48 h	0.37 ± 0.21^*∗*^	0	5.66 ± 1.39^*∗*^	2.54 ± 1.59^*∗*^
BM-Mph-BCG-4 h	0.34 ± 0.25	0	1.16 ± 0.89	4.29 ± 1.48
BM-Mph-BCG-24 h	0.4 ± 0.26	0	3.39 ± 1.81	4.58 ± 3.34
BM-Mph-BCG-48 h	3.88 ± 1.68^*∗*^	0	8.39 ± 1.54^*∗*^	9.0 ± 1.75^*∗*^
Per-Mph-20 d (*n* = 2, 72 h)	11.32 ± 3.31	0	8.11 ± 1.53	3.68 ± 1.16
Gran/20 d (*n* = 2, 72 h)	41.85 ± 2.0^*∗*^	0	96.5 ± 1.23^*∗*^	26.54 ± 1.23^*∗*^
Gran/1 m (*n* = 3, 72 h)	17.69 ± 3.15^*∗*^	77.3 ± 4.68	17.39 ± 1.41^*∗*^	13.78 ± 1.01
Gran/2 m (*n* = 5, 48–96 h)	38.87 ± 1.89^*∗*^	21.59 ± 1.91	23.23 ± 1.03^*∗*^	25.28 ± 0.92^*∗*^
Gran 14/2 m (*n* = 1, 120 h)	7.41 ± 0.91	1.54 ± 0.92	ND	ND

Data are presented as the mean percentage of the number of cytokine-positive cells out of the macrophage population in the granulomas from different mice or in the cultures of mouse peritoneal and bone marrow macrophages, and the standard error of the mean. In each experiment, more than 1000 cells were analyzed at each time point. ^*∗*^ *P* < 0.05 (comparisons of cytokine-positive macrophages in each Gran group and BM-Mph-BCG-48 h, BM-Mph-control-48 h); ND, not done (no staining was performed).
